# Connecting dots of long COVID-19 pathogenesis: a vagus nerve- hypothalamic-pituitary- adrenal-mitochondrial axis dysfunction

**DOI:** 10.3389/fcimb.2024.1501949

**Published:** 2024-12-13

**Authors:** Marta Camici, Giulia Del Duca, Anna Clelia Brita, Andrea Antinori

**Affiliations:** ^1^ Clinical and Research Infectious Diseases Department, National Institute for Infectious Diseases Lazzaro Spallanzani Istituto di Ricovero e Cura a Carattere Scientifico (IRCCS), Rome, Italy; ^2^ Department of Clinical Psychology, National Institute for Infectious Diseases Lazzaro Spallanzani Istituto di Ricovero e Cura a Carattere Scientifico (IRCCS), Rome, Italy

**Keywords:** long COVID, vagus nerve dysfunction, hypothalamic-pituitary-adrenal axis reflex, cholinergic anti-inflammatory reflex, adrenergic anti-inflammatory reflex, cortisol, glucocorticoids receptor, mitochondrial dysfunction

## Abstract

The pathogenesis of long COVID (LC) still presents many areas of uncertainty. This leads to difficulties in finding an effective specific therapy. We hypothesize that the key to LC pathogenesis lies in the presence of chronic functional damage to the main anti-inflammatory mechanisms of our body: the three reflexes mediated by the vagus nerve, the hypothalamic-pituitary-adrenal (HPA) hormonal axis, and the mitochondrial redox status. We will illustrate that this neuro-endocrine-metabolic axis is closely interconnected and how the SARS-CoV-2 can damage it at all stages through direct, immune-inflammatory, epigenetic damage mechanisms, as well as through the reactivation of neurotropic viruses. According to our theory, the direct mitochondrial damage carried out by the virus, which replicates within these organelles, and the cellular oxidative imbalance, cannot be countered in patients who develop LC. This is because their anti-inflammatory mechanisms are inconsistent due to reduced vagal tone and direct damage to the endocrine glands of the HPA axis. We will illustrate how acetylcholine (ACh) and cortisol, with its cytoplasmatic and cellular receptors respectively, are fundamental players in the LC process. Both Ach and cortisol play multifaceted and synergistic roles in reducing inflammation. They achieve this by modulating the activity of innate and cell-mediated immunity, attenuating endothelial and platelet activation, and modulating mitochondrial function, which is crucial for cellular energy production and anti-inflammatory mechanisms. In our opinion, it is essential to study the sensitivity of the glucocorticoids receptor in people who develop LC and whether SARS-CoV-2 can cause long-term epigenetic variations in its expression and function.

## Introduction

Long COVID (LC) was redefined by the US National Academies of Sciences, Engineering, and Medicine as “an infection-associated chronic condition that occurs after severe acute respiratory syndrome Coronavirus 2 (SARS-CoV-2) infection, present for at least three months, manifesting as continuous, relapsing, or progressive disease affecting one or more organ systems” (A Long COVID Definition: A Chronic, Systemic Disease State with Profound Consequences, 2024). This consensus classified LC among chronic health issues following infections of any kind, such as myalgic encephalomyelitis/chronic fatigue syndrome (ME/CFS) and Lyme-associated chronic illness, without needing laboratory confirmation of the initial infection (A Long COVID Definition: A Chronic, Systemic Disease State with Profound Consequences, 2024). LC can worsen preexisting conditions or emerge as new issues, impacting individuals’ ability to work, attend school, care for families, and manage self-care. A recent meta-analysis found that nearly half of Coronavirus disease 2019 (COVID-19) survivors reported lingering symptoms around 120 days post-recovery, with about almost 7.3% developing high-morbidity LC, predominantly affecting young, productive individuals (Mastrorosa et al., 2023). In minors, LC is linked to a more severe acute illness requiring hospitalization, whereas in adults, it often occurs in those with mild to moderate COVID (Su et al., 2022). Risk factors for developing LC include female sex (Bai et al., 2022), multimorbidity, unvaccinated status (Robertson et al., 2023), EBV reactivation, autoimmunity disorders, asthma, type 2 diabetes (Su et al., 2022), attention deficit hyperactivity disorder (ADHD), chronic urticaria and allergic rhinitis (Merzon et al., 2022), although a third of people with LC have no identified pre-existing conditions (Davis et al., 2023). Unfortunately, no biological marker for LC has been found, and no cures exist, with spontaneous recovery being rare (Ely et al., 2024). Actually, various pathogenetic mechanisms of LC have been proposed, including persistent infection, autoimmunity, antigenic mimicry, mitochondrial damage, vagus nerve (VN) injury (Woo et al., 2023), hypercoagulability, microbiota alterations (Alvarez-Santacruz et al., 2024), neurotropic virus reactivation, hypothalamic-pituitary-adrenal (HPA) gland axis dysfunction, and epigenetic modification in gene cell expression (Cheong et al., 2023). Different clinical form of LC have been identified, based on symptoms, but it remains unclear if they reflect distinct pathogenetic pathways. SARS-CoV-2 infection likely has a pleiotropic effect, with multiple causative pathways present concurrently. Notably, LC symptoms fluctuate unpredictably, and the hierarchy and relationships between these mechanisms are still not fully understood, nor is it clear if a common thread connects them.

## Vagus nerve damage may play a central role in long COVID pathogenesis, potentially reducing the body’s anti-inflammatory response and mitochondrial cell function

Our hypothesis proposes that VN damage is a primary contributor to LC development, leading to dysautonomia and disruption of key anti-inflammatory pathways, and mitochondrial function (VanElzakker, 2013). SARS-CoV-2 has been shown to induce both direct and indirect VN damage (Andersson and Tracey, 2024). Woo et al. conducted a post-mortem analysis demonstrating direct SARS-CoV-2 infection of the VN, accompanied by significant neuroinflammation (Woo et al., 2023). Specifically, by studying cells gene expression, they observed increased interferon (IFN) signaling in activated monocytes, glial cells, endothelial cells, and Schwann cells within brain tissue, irrespective of viral load (Woo et al., 2023). Conversely, SARS-CoV-2 RNA load in vulnerable neurons showed a positive correlation with upregulation of stress response mechanisms (e.g., autophagy, proteasomal breakdown). However, these higher levels of intracellular SARS-CoV-2 RNA were also associated with reduced activity in genes responsible for neurotransmitter signaling and neuronal transport, showing a dose-dependent direct VN damage and a dose independent neuroinflammatory response (Woo et al., 2023). Moreover, autoantibodies targeting receptors involved in the vagal anti-inflammatory reflex have been found in convalescent COVID-19 patients, indicating potential functional impairment beyond nerve damage (Dobrowolska et al., 2023). In addition to the VN impairment mechanisms already mentioned, alterations in coagulation and the reactivation of herpes viruses can collaborate to neurotoxicity (VanElzakker, 2013). Interestingly, VN dysfunction triggered by SARS-CoV-2 infection occurs early on during the infection and contributes to the virus’s virulence. In the acute phase, the impairment of the anti-inflammatory reflex may sustain the development of severe cytokine storms, leading to conditions like Acute Respiratory Distress Syndrome (ARDS) and micro embolisms, which increase organ damage and worsen prognosis severity (Johnston and Webster, 2009; Song et al., 2020). Accordingly, Woo et al. found that COVID-19 patients who died exhibited a lower respiratory rate compared to survivors, even in the presence of elevated blood carbon dioxide and severe respiratory insufficiency. This finding suggests that damage to the VN, resulting in autonomic dysfunction, may contribute to respiratory failure in severe COVID-19 cases (Woo et al., 2023).

In the post-COVID phase, impaired vagal signaling could have a pivotal role in preventing the body from restoring inflammatory balance, ultimately perpetrating the LC syndrome (Llados et al., 2024). To strengthen our position, this mechanism was already postulated in a cohort of patients affected by irritable bowel syndrome (IBS) and Crohn’s disease (CD) (Pellissier et al., 2014). In fact, this relevant research found that healthy individuals with higher vagal tone in the morning, defined as an high heart rate variability (HRV), tended to have lower cortisol levels in the evening (Pellissier et al., 2014). This inverse relationship proves the clear neuroendocrinal correlation between VN and axis and suggests that greater vagal tone is associated with better regulation of the stress hormone cortisol (Pellissier et al., 2014). Interestingly, this correlation was not observed in patients with IBS, suggesting an impairment in the vagal anti-inflammatory mechanisms. Further supporting this finding, lower vagal tone in CD patients correlated with higher levels of the inflammatory marker tumor necrosis factor-alpha (TNF-α) (Pellissier et al., 2014). The same study underlined that IBS patients with low vagal tone display high epinephrine levels, indicating heightened and potentially maladaptive sympathetic nervous system activity (Pellissier et al., 2014). Accordingly, HRV was found to be reduced in LC patients compared to a control group, thus reflecting a reduced VN tone (da Silva et al., 2023).

**Figure 1:**
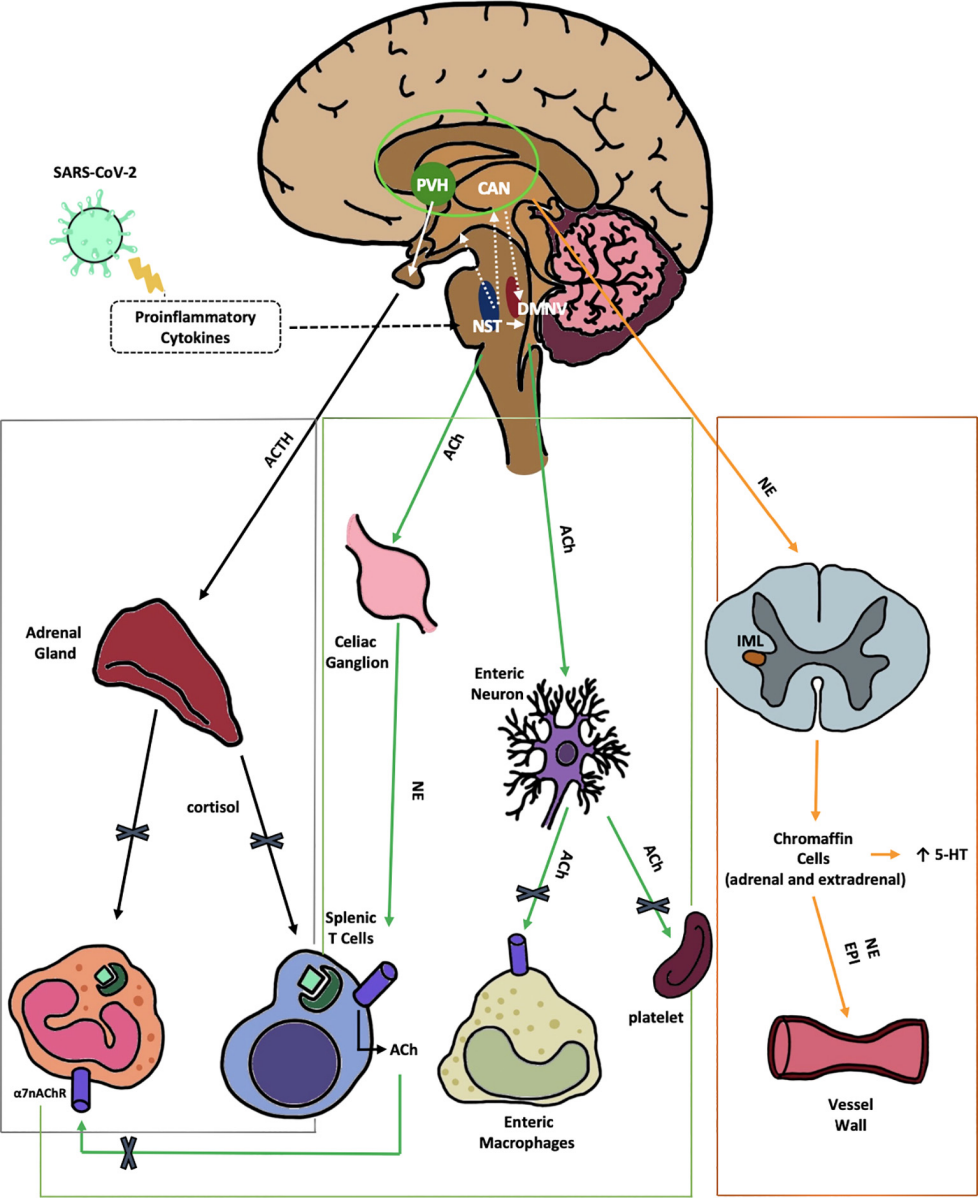
Anti-inflammatory pathways of the VN disrupted by SARS-CoV-2. 1) The HPA axis reflex (black) depicts the suppression of the hypothalamic-pituitary-adrenal axis reflex, leading to a reduction in cortisol production by SARS-CoV-2. This reduction in cortisol impairs the regulation of both innate and adaptive immune responses. Nerve fibers from the NTS stimulate the release of CRH by neurons in the PVH, a hypothalamic nucleus within the central autonomic network. The CAN, comprised of the thalamus, amygdala, hypothalamus, and brainstem nuclei, integrates emotional, sensory, and cognitive stimuli to produce autonomic and endocrine responses. 2) The cholinergic anti-inflammatory reflex (green) illustrates the VN projection to the gastrointestinal tract. This reflex is initiated by afferent fibers from the NTS, which relay peripheral visceral sensory information to the DMNV. ACh, released from DMNV efferent fibers, finally inhibits cytokine release from intestinal macrophages, thereby mitigating local inflammation. Moreover ACh suppress platelets activation. 3) The vago-sympathetic pathway (orange) is activated by vagal efferent stimuli originating from the NTS. This pathway regulates sympathetic outflow, thought the CAN. It targets preganglionic sympathetic neurons in the IML of the spinal cord and chromaffin cells. This complex network modulates peripheral blood tone, vasoconstriction, and immune responses. Enterochromaffin cells are involved in the gut metabolism of 5-HT. α7-nAChR, αlpha7-nicotinicACh-Receptor; ACh, acetylcholine; ACTH, adrenocorticotropic hormone; CAN, central autonomic network; DMNV, dorsal motor nucleus of vagus nerve; EPI, epinephrine; HPA, hypothalamic–pituitary–adrenal; IML, intermediolateral nucleus; NE, norepinephrine; NTS, nucleus tractus solitarius; PVH, parvo-cellular nucleus; VN, vagus nerve; 5-HT, serotonin.

The VN plays a crucial role in regulating the anti-inflammatory response and maintaining balance within the neuro-endocrine-immune system. This is achieved by stimulating the nucleus of the solitary tract (NTS) in the medulla oblongata. The NTS, activated by afferent VN fibers, detects and responds to inflammatory and immune signals from body tissues, initiating three separate pathways (**Figure 1**) (Kaniusas et al., 2020).

## The hypothalamic-pituitary-adrenal axis reflex: the neuroendocrine anti-inflammatory pathway

The VN carries out its anti-inflammatory function by activating the axis reflex, leading to increased cortisol production. Therefore, it plays a crucial role in the neuro-immuno-endocrine axis (**Figure 1**, Black line).

The efferent A2 noradrenergic group of VN fibers from the NTS directly activate the secretion of corticotrophin-releasing hormone (CRH) by the hypothalamic neurons located in the parvo-cellular PVH nucleus (Kaniusas et al., 2020). This nucleus plays a role within the central autonomic network (CAN), an intricate network connecting various nervous centers such as the thalamus, amygdala, hypothalamus, and brain stem nuclei. Together, these centers integrate emotional, sensory, and cognitive stimuli to generate autonomic, behavioral, and endocrine responses. Upon CRH stimulation, adrenocorticotropic hormone (ACTH) is released from the pituitary gland. ACTH then travels to the adrenal glands where it prompts the production of cortisol. Cortisol, in turn, hinders the activation of both innate immunity (splenic and tissue macrophages) and adaptive immunity (spleen T-lymphocytes) via interaction with the glucocorticoid receptor (GR). Glucocorticoids (GCs) bind to the cytoplasmic GR, facilitating its translocation into the nucleus as a transcription factor (Scheinman et al., 1995). A neurovisceral integration model suggests that impaired VN tone, frequently seen in LC patients (Acanfora et al., 2022), contributes to chronic changes in cortisol production and elevated levels of proinflammatory cytokines and acute-phase proteins (Thayer and Sternberg, 2006).

GCs reduce inflammation in several ways. One key mechanism is increasing the production of IKBα protein, which blocks the activity of nuclear factor kappa B (NF-KB), a factor that activates immune response genes (Auphan et al., 1995). GCs also impact immune cells like monocytes, macrophages, and T helper (Th) lymphocytes, influencing their movement, function, and survival (Quatrini and Ugolini, 2021). For example, GCs can suppress inflammation in asthma by altering the balance of Th1 and Th2 cells (Hu et al., 2018). Additionally, GCs can increase CXCR4 expression, affecting the migration of B cells (Cain et al., 2020). Recently, it was discovered that GCs can also activate immune checkpoints in cytotoxic lymphocytes, further suppressing immune responses (Quatrini et al., 2018).

During SARS-CoV-2 infection, both vagal signaling and axis function appear to be impaired, potentially hindering this reflex at various stages. A comparison of autopsies from individuals who died from COVID-19 and those who died from influenza revealed that all COVID-19 patients, but only a small number of influenza patients, had adrenalitis with significantly more severe damage to the structure of the adrenal cortex. Additionally, COVID-19 patients with intensive care unit (ICU) stays exceeding one week showed widespread fibrosis and degeneration of their adrenal glands (Paul et al., 2022). The same authors demonstrated that the SARS-CoV-2 virus has a strong affinity for and replicates effectively *in vivo* within adrenal cortical cells, which express angiotensin converting enzyme 2 (ACE2) and transmembrane protease serine 2 (TMPRSS2) membrane receptors (Paul et al., 2022). Intriguingly, a UK study on 353 hospitalized patients with suspected COVID-19 found that those with confirmed infections had significantly higher cortisol levels early in their illness compared to those without COVID-19 (Tan et al., 2020). This suggests potential adrenal injury and a strong stress response in COVID-19 patients. Additionally, the high cortisol levels was a reliable severity disease marker during the acute infection (Tan et al., 2020). The authors propose that these patients may develop adrenal insufficiency later in their illness, similar to what has been seen in patients with prolonged ICU stays (Tan et al., 2020). Accordingly, impairment of the VN neuro-endocrine reflex, coupled with potential direct damage to endocrine glands by SARS-CoV-2, can lead to reduced cortisol secretion, that was an hallmark of LC (Klein et al., 2023). Notably, a US study identified low morning blood cortisol as a strong predictor of LC development (Wallukat et al., 2021; Klein et al., 2023). This study also observed a blunted stress-induced increase in ACTH and a flat diurnal rhythm of GCs in LC patients, suggesting impaired axis feedback regulation (Jacobson et al., 1988) as previously observed for SARS-CoV (Leow et al., 2005). Indeed, after the acute phase of SARS-CoV, many survivors develop central hypocortisolism, which is characterized by low or inappropriately normal ACTH levels (Leow et al., 2005).

It is also fascinating that molecular mimicry between ACTH and the SARS-CoV viruses has been shown (Wheatland, 2004). The presence of antibodies against SARS-CoV-2 could collaborate to impair the body’s stress response and potentially impact the cortisol response during LC (Wheatland, 2004). Even more interesting is the fact that cortisol seems to directly inhibit the binding of the Spike S1 protein to its intracellular ACE2 receptor, a mechanism that has been postulated to underlie asymptomatic infections (Sarker et al., 2022). The central role of adrenal dysfunction in both LC and ME/CFS is further underscored by the significant overlap of symptoms with adrenal fatigue (AF). AF, a stress-related disorder, typically arises when the adrenal glands struggle to meet heightened cellular energy demands following prolonged stress or trauma (Wilson, 2014). While the similarities in clinical presentation are striking, unlike LC, AF can often be reversed through significant lifestyle modifications, dietary adjustments, and supplements, although recovery may take up to two years (Wilson, 2014).

## The cholinergic anti-inflammatory reflex is fundamental in maintaining anti-inflammatory and coagulative homeostasis

The second anti-inflammatory reflex mediated by the VN, the cholinergic anti-inflammatory reflex (**Figure 1**, green lines), involves efferent fibers from the dorsal motor nucleus of the VN (DMNV). This reflex is initiated by afferent fibers from the NTS, which relay peripheral visceral sensory information to the DMNV (Kaniusas et al., 2020). The DMNV, through its efferent cholinergic pathway, modulates immune responses in the spleen (via the celiac ganglion), liver, and gastrointestinal (GI) tract by the enteric neurons suppressing pro-inflammatory cytokines. In the spleen, acetylcholine (ACh) stimulates celiac neurons, leading to norepinephrine (NE) release, which suppresses cytokines from macrophages, both directly and via splenic T-cell activation. Similarly, ACh stimulates cholinergic enteric neurons in the GI tract, locally inhibiting the innate immune response (Pavlov and Tracey, 2012). Research in mice shows that absence of this reflex enhances innate immune responses and cytokine-related toxicity (Auphan et al., 1995). Moreover, animal models inducing sepsis, ischemia-reperfusion, and pancreatitis, have indicated that VN nerve stimulation (VNS) reduces TNF-α synthesis, prevent acute inflammation, and ultimately enhance overall health outcomes (Borovikova et al., 2000). This reflex involves ACh receptors (AChR) in the celiac ganglion, α7-nicotinic acetylcholine receptors (α7-nAChR) on macrophages, and both NE receptors and β2-adrenergic receptors (β2AR) on T-lymphocytes and macrophages in the spleen. In α7-subunit knockout mice, TNF-α levels fail to decrease after endotoxin exposure, along with a less significant reduction in interleukin(IL)-1β and IL-6 production (Wang et al., 2003), highlighting the receptor’s role in reducing inflammation. T lymphocytes, central to both effector and regulatory immune functions, play a critical role in inflammation and autoimmunity (Hu et al., 2018). Notably, distinct splenic and intestinal T cell subsets express functional choline acetyltransferase (ChAT) and synthesize ACh, constituting a population termed ChAT+ T cells. These ChAT+ T cell subsets are predominantly localized near catecholaminergic splenic nerve fibers, establishing a non-neuronal cholinergic reservoir. The ACh released within this microenvironment activates α7-nAChR on T cells, thereby facilitating their activation and proliferation (Halder and Lal, 2021). Nicotine activation of α7-nAChR in a mouse model of autoimmune encephalomyelitis alleviated symptoms, shifting CD4+ T cells tow an anti-inflammatory IL-4-producing Th2 phenotype while reducing Th1 and Th17 cytokines (Yamakawa et al., 2020). Intriguingly, a sequence in the SARS-CoV-2 spike glycoprotein receptor-binding domain (RBD), termed SARS-CoV-2 glycoprotein peptide (SCoV2P), shares homology with the snake venom neurotoxin NL1, that interact with nAChRs (Farsalinos et al., 2020). This peptide, SCoV2P, modulates α7-nAChRs, enhancing ACh-mediated currents at low concentrations and inhibiting them at higher concentrations (Farsalinos et al., 2020). Other immune cells like mast cells, microglia cells, Kupffer cells may also express α7-nAChRs and could potentially be sensitive to ACh’s anti-inflammatory effects (Wang et al., 2003). Moreover, α7-nAChRs on platelets form functional Ca2+ channels, suggesting that ACh acts as a natural inhibitor of platelet activation (Schedel et al., 2011), with implications for thrombotic complications in COVID-19. Li et al. demonstrated that chronic VN stimulation in mice can suppress endothelial activation during inflammation (Li et al., 2016). Further research by the same group revealed that Ach, by binding nAChRs, inhibits the expression of adhesion molecules like VCAM-1, ICAM-1, and E-selectin on endothelial cells. This binding also reduces cytokine production, providing new insights into the anti-inflammatory and anti-thrombotic effects associated with the cholinergic anti-inflammatory reflex (Li et al., 2022). Supporting this, some LC patients experienced improvement with nicotine patches, which act as competitive agonists for nAChRs, potentially restoring cholinergic function (Leitzke, 2023).

## The vagus-sympathetic pathway influences the anti-inflammatory reflex and regulates serotonin production and reabsorption in the gut

The vagus-sympathetic pathway is the third autonomic route potentially impaired in LC. Vagal efferent stimuli from the NTS activate five brain nuclei within the CAN, which regulates sympathetic outflow and targeting preganglionic sympathetic neurons located in the intermediolateral nucleus (IML) of the spinal cord (Woo et al., 2023) (**Figure 1**, orange line). This network modulates blood tone, vasoconstriction, and immune responses through postganglionic sympathetic neurons targeting adrenal chromaffin cells, enterochromaffin cells (ECs), the celiac ganglion, and the spleen (Andersson and Tracey, 2024). Challenging the traditional view of the VN as purely parasympathetic, experimental studies has shown that in many species the VN stimulation has a double role in both increasing and reducing heart rate (Chiang and Leaders, 1966), and allow to retain endogenous NE in heart (Jellinek et al., 1965). Finally, an animal histochemical study identified a component of adrenergic fibers in the VN, which are characterized as small and unmyelinated (Muryobayashi et al., 1968). This suggests the VN may influence the anti-inflammatory reflex also by modulating sympathetic activity.

Interestingly, a recent study found that LC is associated with decreased enteral reabsorption of serotonin (5-HT), potentially due to gut inflammation and impaired vagal signaling, both mediated by SARS-CoV-2 (Wong et al., 2023). While primarily stored in ECs, 5-HT is also present in gut nerve terminals and mast cells. The vagal-adrenergic pathway is essential for 5-HT release from ECs into portal circulation and the gut lumen, mediated by sympathetic fibers (Linan-Rico et al., 2016). Furthermore, gut 5-HT has been shown to upregulate VN activity, demonstrating a bidirectional relationship. Intact vagal signaling between the gut and brain is essential for mediating the behavioral effects of orally administered selective 5-HT reuptake inhibitors (SSRIs) (McVey Neufeld et al., 2019). This suggests that SSRIs may function, in part, by restoring VN function, highlighting its crucial in 5-HT regulation (McVey Neufeld et al., 2019). According to this theory, while intestinal inflammation may contribute to reduced 5-HT absorption, the inability to restore efficient VN signaling could be a key factor in the 5-HT depletion observed in LC. Peripheral 5-HT deficiency, in turn, impairs cognitive function via reduced vagal signaling (Manta et al., 2009; McVey Neufeld et al., 2019). Given this understanding, therapeutic strategies targeting the VN hold promise for LC treatment. Preliminary studies indicate that non-invasive VNS may improve cognitive function, mood, sleep, and fatigue in LC patients (Yap et al., 2020; Zheng et al., 2024).

## Autoantibodies targeting G-protein-coupled receptors may play a pivotal role in altering neurological, immunological, and cellular functions in long COVID

The impairment of the three identified anti-inflammatory reflexes appears central to the pathogenesis of LC. This impairment may stem from initial direct VN damage, perpetuated by a dysregulated host response to SARS-CoV-2 remnants, particularly in the gut. This sustained immune response could lead to the production of autoantibodies targeting receptors crucial to these reflexes and vasal nerve tone. Intriguingly, a recent preprint demonstrated that transferring IgG from specific LC patient subgroups, stratified by symptom patterns, to healthy mice induced similar LC symptoms. This finding aligns with previous research in ME/CFS and fibromyalgia (Blomberg et al., 2018; Goebel et al., 2021; Chen et al., 2024).

This theory is supported by the observation of elevated circulating autoantibodies in LC patients compared to healthy individuals. Many of these autoantibodies target GPCR muscarinic (mAChR) or nAChR and adrenergic receptors (Wallukat et al., 2021). While the precise pathogenic mechanisms of anti-GPCR autoantibodies in LC warrant further investigation, these autoantibodies could contribute to LC pathology by causing damage to nerve signal transmission, reducing microvascular blood flow, directly impacting cardiac tissue, and/or triggering mast cell activation. Given the established roles of anti-GPCR autoantibodies in diseases like glaucoma and cardiomyopathy, and the promising efficacy of GPCR-targeting molecules in preclinical studies (Campbell and Smrcka, 2018), further research is warranted to elucidate the interplay between autoimmunity, GPCR dysfunction, and the development of LC.

## Mitochondrial dysfunction in SARS-CoV-2 acute infection and long COVID

Mitochondria generate adenosine triphosphate (ATP), the primary energy currency of cells, through cellular respiration and are crucial for cells function. Additionally, mitochondria contribute to various cellular processes, including cell differentiation, autophagy, apoptosis, calcium 2+ (Ca2+) signaling, and thermoregulation (Andrieux et al., 2021). This double-membrane organelles contain multiple copies of their own DNA (mtDNA), that encode subunits of the respiratory-chain complexes, as well as ribosomal and transfer RNAs needed for their synthesis (Wallace, 2005). However, complex II subunit and other mitochondrial proteins, are encoded by the nuclear genome and transported into the mitochondria, where they fold into their functional forms (Walker and Moraes, 2022). Under stressful events, if the mechanisms ensuring mitochondrial integrity and protein folding fail, misfolded proteins can accumulate within mitochondria, leading to dysfunction and cellular apoptosis (Tatsuta, 2009). The dynamic nature of mitochondria allows them to adapt their mass based on cellular energy demands and external stimuli (Scarpulla et al., 2012). They constantly undergo fission and fusion to maintain cellular homeostasis and eliminate damaged organelles through mitophagy (Twig et al., 2008). Notably, mitochondria play a vital role in the innate immune and inflammatory responses. In fact, upon detecting infection, pattern-recognition receptors (PRRs) starts mitochondrial antiviral signaling proteins, leading to increased production of mitochondrial reactive oxygen species (mtROS) (Andrieux et al., 2021). This process activates the Nucleotide-binding oligomerization domain, Leucine rich Repeat and Pyrin domain containing 3 (NLRP3) inflammasome, induces pro-inflammatory gene expression, and triggers the release of pro-inflammatory cytokines, ultimately leading to the elimination of infected cells and viral clearance (Mantle et al., 2024).

Although mitochondrial damage is a common virulence mechanism for many pathogens to evade the immune response (Mehrzadi et al., 2021; Singh et al., 2021), animal models have confirmed that coronaviruses (CoVs) are particularly pernicious against them, directly invading mitochondria and relying on them for its replication (Zhang et al., 2020). A study, comparing gene expression in lung cells infected with Influenza A virus, SARS-CoV-2, and Middle East respiratory syndrome (MERS)-CoV, revealed that all three viruses caused an increase in IFN signaling genes. However, only the two CoVs caused increment of mtROS production and perturbation in autophagy. Besides, only SARS-CoV-2 infection led to an increase in inflammatory and cytokine signaling genes (Singh et al., 2021). SARS-CoV-2 has been shown to manipulate host cell mitochondria for its benefit, inducing mitochondrial damage at multiple levels of biological function. The mitochondrial mechanisms damage, during SARS-CoV-2 infection, are listed in **Table 1**. For instance, the spike protein can reduce normal mitochondrial respiration and ATP production while simultaneously increasing glucose-induced glycolysis to promote its metabolic path (Lei et al., 2021; Shang et al., 2021). This effectively hijacks the host’s metabolic processes, as evidenced by elevated lactate and glucose levels in infected patients (Moolamalla et al., 2021). Studies show that the virus disrupts the organization and function of the electron transport chain, as evidenced by the mis-localization of key proteins in infected cells (Soria-Castro et al., 2021). This disruption, along with impaired expression of mitochondrial and antioxidant genes, compromises the mitochondrial membrane and the oxidative phosphorylation (OXPHOS), ultimately increasing harmful mtROS (Soria-Castro et al., 2021). Furthermore, to improve its replication, SARS-CoV-2 directly downregulate the expression of genes crucial for mitochondrial ribosomes and Complex I, thereby impairing the mitochondrial respiratory chain, increasing cell oxidative stress causing the loss of mitochondrial integrity and cell death (Singh et al., 2021). Another mitochondrial injury mechanism, is mediated by the CoVs non-structural proteins Open Reading Frame (ORF), such as ORF-7a, ORF-8a and ORF-9b, which are located in mitochondria and inhibit the retinoic acid-inducible gene I-mitochondrial antiviral signaling protein (RIG1-MAVS)-dependent IFN signaling, evading host cell immunity and promoting viral replication (Singh et al., 2020). Additionally, ORFs can directly trigger the mitochondrial apoptotic pathways (Ren et al., 2020; Mehrzadi et al., 2021). Specifically, the SARS-CoV-2 ORF3a protein can dimerize and generate a non-selective Ca2+ permeable cation channels on the mitochondrial membrane, that upregulates Ca2+ signaling from endoplasmic reticulum (Davies et al., 2020). This activates e cascade starting with Ca2+-dependent caspases, that leads to programmed cell death (Ren et al., 2020), contributing to the reduced lymphocyte count observed in COVID-19 (Ren et al., 2016; Mehrzadi et al., 2021). SARS-CoV-2 proteins can disrupt cellular Ca2+ balance also by direct blocking L-type Ca2+ channels, perturbing cardiac energetic and ultimately cause cardiomyocyte death (Ramachandran et al., 2022). Moreover, SARS-CoV-2 may induce the NLRP3 inflammasome, and upregulate the expression of the inflammatory cytokine genes such as IL-1β and IL-18 and contributing to the proinflammatory storm. This can trigger pyro-ptosis, a highly inflammatory form of programmed cell death, particularly in lymphocytes and macrophages (Yang, 2020). Additionally, CoVs can antagonize the unfolded protein response and organelle fission, resulting in hyperfused, non-active mitochondria (Tondera et al., 2009; Shi et al., 2014). Accordingly, a study on mitochondrial morphological changes during acute infection found mitochondrial swelling and vacuolization after one day (Shang et al., 2021). Notably, a recent study has shown that the degrading mitochondrial material eliminated by the cells stolen in extracellular vesicles can activate the innate immune response and autoantibodies cross reaction against mitochondrial antigens (Di Florio et al., 2024). In addition to that, SARS-CoV-2 has been shown to disrupt mitophagy. While the virus initially upregulates this process by activating the Pink1-Parkin-P62 pathway, it subsequently inhibits its completion by obstructing the binding of P62 and LC3, which is essential for the selective engulfment of targeted components into autophagosomes (Shang et al., 2021). Lastly, SARS-CoV-2 may indirectly affect mitochondrial function by impacting cortisol and Ach metabolism, as discussed later.

**Table 1:** Mitochondrial Damage Mechanisms Induced by SARS-CoV-2.

Author	SARS-CoV-2 mediators	Mechanism	Outcome
**Cao et al. (** Cao et al., 2023)	Spike protein	Suppression of respiratory chain genes including ATP synthases	Impaired ATP production
**Di Florio et al. (** Di Florio et al., 2024)	SARS-CoV-2	Mitochondria extracellular vesicle	Mitochondria auto-Ab
**Ley et al. (** Lei et al., 2021)	Spike protein	ACE2 downregulation	↑ Endothelial glycolysis
**Ramachandran et al. (** Ramachandran et al., 2022)	M-protein, NSP6, ORF3A, ORF9C, ORF10	Inhibition of L-type Ca2+ channels	Cell apoptosis
**Ren et al. (** Ren et al., 2020)	ORF-3	Alteration of Ca2+ mitochondrial signaling	Cell apoptosis
**Sci et al. (** Shi et al., 2014)	ORF-9b	Degradation of DRP1	Hyperfused mitochondria
**Shang et al. (** Shang et al., 2021)	SARS-CoV-2	Inhibition of P62 and LC3 binding	↓ Mitophagy
**Singh et al. (** Singh et al., 2021)	SARS-CoV-2	Complex I gene downregulation	Impaired ATP production
**Singh et al. (** Singh et al., 2020)	ORF-7a, ORF-8a, ORF-9b	Inhibition of RIG1-MAVS dependent IFN signaling	To evade host cell immunity and viral induce replication
**Soria-Castro et al. (** Soria-Castro et al., 2021)	M-protein, NSP2, NSP7	OXPHOS impairment	↑ mtROS
**Yang et al. (** Yang, 2020)	PAMPS	Induction of NLRP3 inflammasome	Activate pyro-ptosis

ATP, adenosine triphosphate; DRP1, dynein-related protein 1 (mitochondrial fission protein); mtROS, mitochondrial reactive oxygen species; LC3, microtubule-associated protein 1 light chain 3; NSP 6, non-structural protein 6; ORF, open reading frame; OXPHOS, oxidative phosphorylation; PAMPs, pathogen-associated molecular patterns; RIG1-MAVS, retinoic acid-inducible gene I-mitochondrial antiviral signaling protein. ↑= increased, ↓= decreased.

Interestingly, mitochondria dysfunction seems last long in those patients developing LC, although the precise mechanisms require further investigation (Chen et al., 2023; Molnar et al., 2024). For example, a study conducted on mice infected by a lentivirus vector encoding sequence of Spike gene demonstrated that Spike protein can induce transcriptional suppression of metabolic genes, determining an impairment in energy production and redox status, hesitating in morphologic cells change, and cardiac fibrosis (Cao et al., 2023). Similarly, neuropsychiatric manifestation of LC resulted correlated with the increment of mitochondrial proteins and SARS-CoV-2 proteins in neuronal and astrocytes-derived patients exosomes (Peluso et al., 2022). Further studies are needed to understand the evolution of mitochondrial damage over time and its specific impact on the pathogenesis of LC. It will also be important to determine the mechanisms by which those who do not develop LC restore basal mitochondrial function and what prevents patients from returning to the previous equilibrium.

## The altered cortisol signaling can affect both cellular gene expression and mitochondrial function

Cortisol, produced in the zona fasciculata cells of the adrenal cortex, is synthesized from cholesterol in mitochondria-rich cells, regulated by two families of mitochondrial enzymes: cytochrome P450 and the hydroxysteroid dehydrogenase/ketosteroid reductase (HSD/KSR) (Midzak and Papadopoulos, 2016).

Although primarily produced in the adrenal gland, extra-adrenal cortisol is also synthesized in the brain, thymus (Talaber et al., 2015), blood vessels, and epithelial cells (Noti et al., 2009), contributing to local hormonal regulation (Taves et al., 2011). Transported in the blood by cortisol-binding protein (Henley et al., 2016), cortisol becomes active inside cells after conversion by 11β-hydroxysteroid dehydrogenase 1 (11β-HSD1), an enzyme with tissue-specific regulation (Timmermans et al., 2019). Biologically active cortisol then binds to its intracellular receptors and mediates a wide range of cell-specific effects (Rhen and Cidlowski, 2005). The human GR gene is highly variable, producing multiple receptor isoforms, with 13 exon-1 variants that regulate transcription, reflecting the complexity of GR gene expression (Timmermans et al., 2019; Quatrini and Ugolini, 2021). The binding of transcription factors to gene variants can significantly affect receptor protein availability in target tissues (Vandevyver et al., 2014), while alternative splicing of the GR gene generates multiple protein isoforms, with intracellular factors further regulating ligand sensitivity. This intricate regulation enables the GR to function in a cell-specific manner, influencing responses to cortisol and GC treatments (Nagy et al., 2016). Epigenetic modifications like DNA methylation and histone acetylation further regulate GR gene expression, which can profoundly influence their function (Timmermans et al., 2019). Interestingly, the presence or absence of specific epigenetic variations has been linked to cancer development and mental health issues (Radtke et al., 2015). Classically, GC/GR controls the expression of around 1,500 genes involved to vital functions including metabolism, cardiac function, immune response, mood, and cognition (Wilson, 2014). However, recent research has uncovered a non-genomic role for GC/GR in mitochondrial function. GC/GR can activate mitochondrial transcription, enhancing cell energy production and regulating electron transport chain function and mtRNA expression in response to stressors (Lapp et al., 2019). While acute corticosteroid stimulation appears to boost mitochondrial oxidation, chronic or excessive stimulation can have the opposite effect (Picard et al., 2018). This is crucial since mitochondria synthesize all steroid hormones, including cortisol, indicating a self-regulating feedback loop (Bose et al., 2002). Progesterone may also act as a stress hormone (Musto and Camici, 2022) and its potential role in SARS-CoV-2 pathogenesis and treatment has to be assessed. As a result, an altered cortisol signaling affects both gene expression and mitochondrial function. Besides, GR and adrenal receptor (AR) are transcriptional activators of ACE2 and the TMPRSS2 both fundamental for the SARS-CoV-2 cells adhesion and penetration. A molecule modulating, through allostatic inhibition its activity, has shown to reduce the severity of SARS-CoV-2 infection in animal models (Rocha et al., 2022). Considering what has already been stated, we believe it is appropriate to evaluate whether the cortisol sensitivity of receptors in patients affected by LC differs compared from those who do not develop it. Additionally, it is important to study the existence of possible epigenetic alterations in the synthesis of this receptor, which is crucial for cellular function, induced by the interaction with the virus.

## Consequences of detrimental cholinergic signaling in long COVID: from neurocognitive impairment to mitochondrial dysfunction

Adding another layer of complexity, a VN dysfunction and the consequent decline in cholinergic signaling can locally contribute to neurodegenerative processes (Chen et al., 2022). As is known, the cholinergic system regulates neurogenesis, synaptic plasticity, neuronal differentiation, and neuroprotection through nAChR and mAChR (Frinchi et al., 2018). Cholinergic neurons in the basal forebrain, rich in ACh-producing fibers, are particularly important for learning, memory, and cognitive function due to their influence on cortical and hippocampal regions (Latina et al., 2018). Experimental models using scopolamine, a muscarinic antagonist, have shown that blocking these receptors can induce Alzheimer Disease (AD)-like pathology by impairing mitochondrial antioxidant systems and increasing reactive oxygen species (Bujan et al., 2019). Conversely, xanomeline, a mAChR (M1/M4) agonist, has shown promise in treating AD and schizophrenia (Montani et al., 2021). Intriguingly, studies have found elevated levels of anti-muscarinic antibodies in the serum of LC and ME/CFS patients compared to controls, further implicating cholinergic dysfunction in these conditions (Wallukat et al., 2021). Notably, ACh synthesis, like cortisol production, depends on mitochondrial function, as ACh is synthesized from acetyl-CoA a byproduct of mitochondrial glycolysis (Halder and Lal, 2021). Further solidifying this link, a study investigating the cardioprotective effects of ACh found that it significantly increased mitochondrial density, mass, and mtDNA copy number, increasing ATP synthesis and mitochondrial activity (Sun et al., 2013). These suggests a feedback loop where ACh both relies on and boosts mitochondrial function, which can be highly impaired in LC.

In summary, these findings highlights the strong biochemical link between vagal and adrenal functions, both disrupted by SARS-CoV-2, and their modulation of mitochondrial activity. Indeed, in a scenario where SARS-CoV-2 infection significantly impairs mitochondria, vagal nerve dysfunction, leading to reduced cholinergic and cortisol signaling, could further hinder mitochondrial function. This could create a vicious cycle, perpetuating oxidative stress, inflammation, cell damage, and ultimately cell death.

## Conclusions

In conclusion, the model of a dysfunctional VN nerve-HPA-mitochondrial axis can provide a comprehensive explanation for the various alterations observed in LC. Disruptions to more components of this intricate system, whether through direct damage, immune dysregulation, autoantibody interference or epigenetic gene modification in predisposed patients may determine the development and the persistence of this complex syndrome. As a consequence, individual susceptibility to LC likely can depends on baseline vagal tone, cortisol sensitivity, basal mitochondrial cell functioning and immunity factors (Dedoncker et al., 2021). According to this theory, individuals with mild pre-existing VN impairment may be more vulnerable to developing LC, particularly when faced with cortisol reductions and epigenetic modifications in GR signaling pathway and mitochondrial genes. Specifically, investigations into the long-term effects of chronically low morning cortisol levels and their impact on the epigenetic regulation of mtDNA will be instrumental, in our opinion, in unraveling the contributions of mitochondria to LC pathogenesis. For example, it has been hypothesized that dysregulation of cellular microRNAs (miRNAs), which are small non-coding RNAs that modulate gene expression by binding to messenger RNA, may contribute to the upregulation of the IL-6/signal transducers and activators of transcription 3 (STAT3) proinflammatory axis, leading to pain in LC patients (Reyes-Long et al., 2023).

With this paper, we aim to highlight the potential fundamental connection between VN dysfunction, the resulting deficit of Ach and cortisol, which directly leads to mitochondrial dysfunction, perpetuating the process and contributing to the exacerbation of LC symptoms. This theory could also partly explain why SARS-CoV infection accelerates aging (Strong, 2023) and facilitates the development of autoimmune diseases (Sharma and Bayry, 2023).

Given this complexity, an integrated and multidimensional approach to patient evaluation is paramount. This evaluation should encompass assessments of VN basal tone, comprehensive autoantibody panels, mitochondrial function, and potentially even genetic typing of relevant receptors and their epigenetic regulation. A thorough understanding of how stress affects mitochondrial physiology requires a multifaceted evaluation that includes measuring mitochondrial copy number, function, and methylation status. An integrated, multidimensional evaluation could enable personalized treatments by targeting simultaneously specific pathways, offering the best hope for addressing LC challenges.

## Data Availability

The raw data supporting the conclusions of this article will be made available by the authors, without undue reservation.
